# Combined Curcumin and Doxorubicin Induce Apoptosis via JNK-Dependent MAPK Signaling Independent of TXNDC5 in Human Osteosarcoma Cells

**DOI:** 10.3390/nu18081235

**Published:** 2026-04-14

**Authors:** Yu-Hsiang Liao, Kai-Chien Yang, Heng-Jing Chen, Ching-Wei Shih, Yi-Cheng Yeh, Jyun-Yu Peng, Fung-Jou Lu, Shang-Tzu Yang, Ching-Hsein Chen

**Affiliations:** 1 Department of Orthopedic Surgery, Ditmanson Medical Foundation Chia-Yi Christian Hospital, No. 539, Zhongxiao Rd., East Dist., Chiayi City 60002, Taiwan; bigxiang54824@gmail.com; 2 Department and Graduate Institute of Pharmacology, College of Medicine, National Taiwan University, No. 1, Jen Ai Road Section 1, Taipei City 100233, Taiwan; kcyang@ntu.edu.tw; 3 Department of Microbiology, Immunology and Biopharmaceuticals, College of Life Sciences, National Chiayi University, A25-303 Room, Life Sciences Hall, No. 300, Syuefu Road, Chiayi City 600355, Taiwan; x311720@gmail.com (H.-J.C.); eason2452@gmail.com (Y.-C.Y.); 4 Institute of Biopharmaceutical Sciences, National Yang Ming Chiao Tung University, No. 155, Sec. 2, Linong Street, Beitou District, Taipei City 112304, Taiwan; a0958798219@gmail.com; 5 Department of Anesthesiology, Chang Gung Memorial Hospital at Chiayi, No. 8, West Section of Jiapu Road, Puzi City, Chiayi County 613016, Taiwan; 6 Institute of Medicine, Chung Shan Medical University, No. 110, Section 1, Jianguo North Road, Taichung City 402306, Taiwan; fjlu@csmu.edu.tw; 7 Department of Hematology/Clinical Microscopy, Cathay General Hospital, No. 280, Renai Rd., Sec. 4, Taipei City 106438, Taiwan

**Keywords:** curcumin, doxorubicin, MAPK, TXNDC5, apoptosis, osteosarcoma

## Abstract

Background: Curcumin, a dietary polyphenol with anticancer potential, has been reported to enhance the efficacy of chemotherapeutic agents. Methods: The effects of combined curcumin and doxorubicin treatment on apoptosis and associated signaling pathways were investigated in human osteosarcoma HOS cells. Results: Combined treatment significantly reduced cell viability and induced apoptotic morphological changes, which were confirmed by increased sub-G1 population, enhanced DNA fragmentation, and elevated cleaved poly(ADP-ribose) polymerase (PARP) levels. Mechanistically, combined treatment markedly increased c-Jun *N*-terminal kinase (JNK) phosphorylation, whereas extracellular signal-regulated kinase (ERK) phosphorylation showed no appreciable change. Pharmacological inhibition revealed that JNK suppression attenuated PARP cleavage, while ERK inhibition also reduced apoptotic responses, suggesting a permissive role of basal ERK activity. In addition, combined treatment was associated with increased expression of the endoplasmic reticulum stress marker GRP78 and modulation of autophagy-associated markers. Although thioredoxin domain-containing protein 5 (TXNDC5) expression was reduced, TXNDC5 overexpression failed to attenuate apoptosis, indicating that apoptosis induction occurs independently of TXNDC5. Conclusions: These findings indicate that combined curcumin and doxorubicin induce apoptosis primarily through JNK-dependent MAPK signaling, accompanied by stress-associated cellular responses.

## 1. Introduction

Osteosarcoma is an aggressive primary bone malignancy for which doxorubicin remains a cornerstone chemotherapeutic agent in both neoadjuvant and adjuvant treatment protocols. Despite its clinical utility, the effectiveness of doxorubicin is frequently compromised by intrinsic or acquired resistance mechanisms in osteosarcoma cells [1,2,3,4]. One well-recognized mechanism involves the overexpression of multidrug transporters such as P-glycoprotein, which actively export chemotherapeutic agents and reduce intracellular drug accumulation [5]. Resistance to platinum-based agents has also been associated with impaired drug uptake, enhanced DNA repair mechanisms, and elevated intracellular antioxidant defenses, including glutathione-mediated detoxification of cytotoxic stress [6,7]. In addition to therapeutic resistance, doxorubicin-induced cardiotoxicity represents a major dose-limiting adverse effect and may lead to progressive cardiomyopathy and heart failure [8]. Over the past three decades, standard combination regimens incorporating methotrexate, doxorubicin, and cisplatin have produced limited improvement in long-term survival, particularly in patients with metastatic disease or poor chemotherapeutic response [9]. These limitations highlight the urgent need for novel strategies that enhance therapeutic efficacy while minimizing systemic toxicity.

Growing interest has focused on dietary phytochemicals and natural compounds as adjuvant agents capable of potentiating chemotherapy while reducing adverse effects [10]. Curcumin, a polyphenolic compound derived from *Curcuma longa*, exhibits diverse biological activities including anti-inflammatory, antioxidant, immunomodulatory, and anticancer properties [11]. Extensive evidence indicates that curcumin exerts antitumor activity through modulation of multiple molecular targets involved in tumor initiation and progression [12,13]. Moreover, curcumin suppresses oncogenic signaling networks while enhancing pro-apoptotic pathways, thereby promoting programmed cell death in cancer cells [14,15]. Owing to its pleiotropic actions and favorable safety profile, curcumin has emerged as a promising candidate for combination therapy aimed at overcoming chemoresistance and improving therapeutic outcomes.

Mitogen-activated protein kinase (MAPK) signaling pathways regulate fundamental cellular processes including proliferation, differentiation, stress responses, and apoptosis [16]. The MAPK family consists primarily of extracellular signal-regulated kinase (ERK), p38 MAPK, and c-Jun *N*-terminal kinase (JNK), each responding to distinct extracellular and intracellular stimuli [17]. Activation of ERK, p38, and JNK has been implicated in apoptosis induction under specific conditions, particularly in response to oxidative stress and chemotherapeutic exposure [18]. Because MAPK pathways integrate stress signals and influence cell fate decisions, they represent attractive therapeutic targets in cancer treatment. Modulation of MAPK signaling has therefore been proposed as a strategy to enhance anticancer efficacy and overcome therapeutic resistance [19,20]. Nevertheless, the relative contribution of individual MAPK branches to chemotherapy-induced apoptosis remains context-dependent and requires further clarification in osteosarcoma.

Thioredoxin domain-containing protein 5 (TXNDC5) has recently attracted attention as an oncogenic factor due to its elevated expression in multiple malignancies and its involvement in tumor progression [21]. TXNDC5 contributes to cancer cell survival by regulating redox homeostasis and cellular stress adaptation. Under hypoxic conditions, TXNDC5 expression is upregulated via hypoxia-inducible factor-1α (HIF-1α), reducing reactive oxygen species–induced endoplasmic reticulum (ER) stress and supporting cancer cell survival [22,23]. Beyond stress adaptation, TXNDC5 has been implicated in cell cycle regulation; its overexpression increases the proportion of cells in the G2/M phase, whereas its suppression results in G0/G1 arrest [21,24]. Functionally, TXNDC5 acts as a protein disulfide isomerase that facilitates protein folding within the ER and protects cells from ER stress-induced apoptosis [22,25]. Elevated TXNDC5 expression has been associated with poor prognosis in several cancers and may serve as a prognostic biomarker [21,22,26]. Inhibition of TXNDC5 has been shown to suppress malignant phenotypes and enhance susceptibility to apoptosis, supporting its potential as a therapeutic target [21,22,23,26]. Our previous work further demonstrated that TXNDC5 suppression enhances chemosensitivity in cancer cells [23].

Given the limitations of doxorubicin therapy and the multifaceted anticancer properties of curcumin, combining these agents represents a rational strategy for osteosarcoma treatment. Curcumin has been shown to sensitize cancer cells to chemotherapeutic agents by modulating pathways associated with survival, oxidative stress, apoptosis, and drug resistance, thereby enhancing anticancer efficacy and potentially allowing dose reduction of cytotoxic drugs [12,27,28,29]. Importantly, curcumin has also been reported to mitigate chemotherapy-induced toxicity in normal tissues, improving the therapeutic index of conventional anticancer therapies [29,30,31,32]. However, the molecular mechanisms underlying the combined effects of curcumin and doxorubicin in osteosarcoma cells remain incompletely defined. Therefore, the present study investigated the effects of combined curcumin and doxorubicin treatment on apoptosis in human osteosarcoma cells and examined the associated signaling pathways, with particular emphasis on MAPK-dependent mechanisms.

## 2. Materials and Methods

### 2.1. Reagents and Chemicals

Minimum Essential Medium (MEM) was obtained from Gibco (Thermo Fisher Scientific, Waltham, MA, USA). Fetal bovine serum (FBS), penicillin–streptomycin, sodium pyruvate, and MEM non-essential amino acids (NEAA) were also purchased from Gibco (Thermo Fisher Scientific, Waltham, MA, USA). Curcumin (from *Curcuma longa* (turmeric), powder form; CAS No. 458-37-7; Sigma-Aldrich, St. Louis, MO, USA) was used in this study. Doxorubicin hydrochloride was acquired from Sigma-Aldrich (St. Louis, MO, USA). Propidium iodide (PI), RNase A, paraformaldehyde (PFA), trypan blue, and routine laboratory reagents were obtained from Sigma-Aldrich. The Cell Counting Kit-8 (CCK-8) assay kit was purchased from Dojindo Laboratories (Kumamoto, Japan), and the Bio-Rad protein assay kit was obtained from Bio-Rad Laboratories (Hercules, CA, USA). X-tremeGENE™ HP DNA transfection reagent was purchased from Roche (Raleigh, NC, USA). MAPK inhibitors PD98059, SB203580, and SP600125 were obtained from Sigma-Aldrich. The TXNDC5 overexpression plasmid and corresponding control vector were obtained from Addgene (Watertown, MA, USA). All other chemicals were of analytical grade. Primary antibodies against cleaved PARP, phosphorylated and total ERK, p38 MAPK, and JNK were obtained from Santa Cruz Biotechnology (Santa Cruz, CA, USA). Anti-TXNDC5 antibody was purchased from Abcam (Waltham, MA, USA). All primary antibodies were used at dilutions of 1:1000 unless otherwise specified. Horseradish peroxidase–conjugated secondary antibodies were used at a dilution of 1:10,000.

### 2.2. Cell Culture

Human osteosarcoma HOS and MG-63 cells were maintained in MEM supplemented with 10% FBS, 100 U/mL penicillin G, 100 μg/mL streptomycin, 250 ng/mL amphotericin B, 1 mM sodium pyruvate, and MEM non-essential amino acids (NEAA). Cells were cultured at 37 °C in a humidified incubator containing 5% CO_2_. Upon reaching approximately 80% confluence, cells were rinsed with phosphate-buffered saline (PBS) and detached using trypsin–EDTA. Enzymatic activity was neutralized with complete medium, and cells were collected by centrifugation at 1000 rpm for 3 min. Cell pellets were resuspended in fresh medium, and viable cells were quantified using trypan blue exclusion and a hemocytometer before seeding at the desired density.

### 2.3. Cell Viability Assay

Cell viability was determined using the CCK-8 assay according to the manufacturer’s protocol. HOS and MG-63 cells were seeded at 4 × 10^4^ cells per well in 24-well plates and allowed to attach for at least 16 h prior to treatment. Cells were treated with curcumin, doxorubicin, or their combination. For curcumin-only treatment, concentrations ranged from 5–20 μM; for doxorubicin alone, concentrations ranged from 25–500 nM. For combination treatment, curcumin (7.5–9 μM) was combined with doxorubicin (0.2 μM). The selected concentration range of curcumin was based on preliminary dose–response experiments to identify sub-cytotoxic conditions (20–40% reduction in cell viability) suitable for evaluating combination effects. Doxorubicin was used as an internal reference control due to its well-established cytotoxic effects in osteosarcoma cells. After 48 h, 20 μL CCK-8 reagent was added to each well and incubated for 1.5 h at 37 °C. Aliquots (200 μL) were transferred to a 96-well plate, and absorbance was measured at 450 nm. Cell viability was expressed relative to untreated controls.

### 2.4. Flow Cytometry Analysis of Sub-G1 Population

Flow cytometric analysis was performed using a CytoFLEX flow cytometer (Beckman Coulter, Brea, CA, USA). Data acquisition was conducted under standard hydrodynamic focusing conditions, and at least 10,000 events were collected per sample. Cell cycle distribution and the sub-G1 population were analyzed by flow cytometry following propidium iodide (PI) staining, which was used as a single-color parameter to determine DNA content. After drug treatment, cells were washed with phosphate-buffered saline (PBS), harvested, and centrifuged at 1300 rpm for 5 min. The supernatant was removed, and the cell pellet was resuspended in 1 mL of PBS. Cells were then transferred to 1.5 mL microcentrifuge tubes. RNase A (4 μL) and propidium iodide (PI; 20 μL) were added to each sample, and the mixtures were incubated for 30 min at room temperature in the dark. All samples were analyzed within 3 h using a flow cytometer to determine cell cycle distribution. The sub-G1 population, defined as a population with DNA content lower than that of the G1 peak, appears to the left of the G1 phase in the DNA content histogram. This population arises from apoptotic cells undergoing DNA fragmentation, resulting in reduced PI incorporation and lower fluorescence intensity. Therefore, the percentage of cells in the sub-G1 fraction was quantified as an indicator of apoptotic cell death.

### 2.5. TUNEL Assay

For detection of apoptotic DNA fragmentation, a terminal deoxynucleotidyl transferase–mediated dUTP nick end labeling (TUNEL) assay was performed using the APO-BrdU™ TUNEL assay kit with Alexa Fluor™ 488 anti-BrdU (Thermo Fisher Scientific; Cat. No. A23210) according to the manufacturer’s instructions. Following labeling, cells were subjected to dual-color staining with Alexa Fluor™ 488–conjugated anti-BrdU and propidium iodide (PI). Alexa Fluor™ 488 fluorescence was used to detect TUNEL-positive cells, while PI staining was used to determine total DNA content. Dual-color flow cytometric analysis was performed to distinguish apoptotic (TUNEL-positive) cells from non-apoptotic populations. Data acquisition and analysis were conducted using appropriate flow cytometry software. A minimum of 10,000 events per sample were collected.

After drug treatment, cells were collected and washed twice with phosphate-buffered saline (PBS) and transferred to 15 mL centrifuge tubes. Cells were then detached by incubation with trypsin at 37 °C in a humidified incubator containing 5% CO_2_ for approximately 3 min. Detached cells were collected, washed with PBS, combined into the same tube, and centrifuged at 1300 rpm for 5 min. The supernatant was carefully removed. Cells were fixed with 5 mL of 1% paraformaldehyde (PFA) at room temperature for 15 min, followed by two washes with PBS to remove residual fixative. Cells were then resuspended in 5 mL of ice-cold 70% ethanol and stored at −20 °C for at least 16–18 h prior to analysis. After fixation and permeabilization, cells were centrifuged at 1300 rpm for 5 min, and the supernatant was removed. Cells were resuspended in 1 mL of wash buffer and centrifuged again at 1300 rpm for 5 min. This washing step was repeated twice to ensure complete removal of residual medium and debris. Subsequently, 50 μL of DNA labeling solution was added to each sample, and cells were incubated at 37 °C for 60 min in a hybridization incubator. During incubation, samples were gently mixed every 15 min to ensure uniform reagent distribution. Following labeling, cells were washed twice with 1 mL of rinse buffer (1300 rpm, 5 min), with complete removal of the supernatant after each wash. Cells were then resuspended in 0.1 mL of antibody solution and incubated at room temperature for 30 min in the dark. Subsequently, 0.9 mL of PI/RNase A solution was added, and samples were further incubated at room temperature for 30 min in the dark. All samples were analyzed by flow cytometry within 3 h of completion of staining. The percentage of TUNEL-positive cells was quantified as an indicator of apoptotic cell death.

### 2.6. Western Blot Analysis

Protein expression levels of cleaved poly(ADP-ribose) polymerase (PARP), mitogen-activated protein kinase (MAPK) signaling proteins, and thioredoxin domain-containing protein 5 (TXNDC5) were analyzed by Western blotting. Protein samples were prepared from whole-cell lysates. After treatment, cells were washed with phosphate-buffered saline (PBS) and lysed in protein extraction buffer on ice for 10 min. The lysates were then centrifuged at 12,000× *g* for 10 min at 4 °C, and the supernatants were collected as total cellular proteins. Protein concentrations were determined using a protein assay reagent (Bio-Rad, Richmond, CA, USA) according to the manufacturer’s instructions. Protein samples were retrieved from −80 °C storage and kept on ice prior to analysis. For experiments performed in HOS cells, four treatment groups were included: untreated control, curcumin-treated (CUR, 9 μM), doxorubicin-treated (DOX, 0.2 μM), and combined curcumin (9 μM) and doxorubicin (0.2 μM) treatment, as indicated in the corresponding figures. For validation experiments performed in MG-63 cells (Supplementary Figure S2), cells were treated with curcumin (15 μM), doxorubicin (0.3 μM), or the indicated combinations for 48 h. Higher concentrations of doxorubicin were selected in MG-63 cells to facilitate clearer detection of apoptotic signaling, as assessed by cleaved PARP expression.

Protein concentrations were determined using a Bio-Rad Protein Assay kit according to the manufacturer’s instructions. Equal amounts of protein (20–30 μg per lane) from each sample were mixed with distilled water and sample buffer to a final volume of 20 μL. Samples were heated for 5 min, immediately placed on ice for 5 min, and briefly centrifuged prior to electrophoresis. Proteins were separated by 12% sodium dodecyl sulfate–polyacrylamide gel electrophoresis (SDS–PAGE) and subsequently transferred onto polyvinylidene difluoride (PVDF) membranes. Membranes were blocked with 0.05% bovine serum albumin (BSA) for 90–120 min at room temperature, followed by washing with phosphate-buffered saline containing 0.1% Tween-20 (PBST) three times for 10 min each. Membranes were then incubated with primary antibodies at 4 °C for 16–18 h. After primary antibody incubation, membranes were washed three times with PBST for 10 min each and incubated with the appropriate horseradish peroxidase–conjugated secondary antibodies at room temperature for approximately 1 h. Membranes were subsequently washed three times with PBST for 10 min each. Protein bands were detected using enhanced chemiluminescence (ECL) reagents, and immunoreactive signals were visualized and quantified using a chemiluminescence imaging system. Membranes were trimmed according to molecular weight markers prior to antibody incubation when appropriate. Each target protein corresponds to the same sample as the loading control, but was obtained from a trimmed membrane processed separately. Band intensities were quantified using image analysis software, normalized to the corresponding loading control (GAPDH or tubulin), and expressed relative to control conditions. Phosphorylation levels were evaluated as relative changes and were not normalized to corresponding total protein levels. All quantitative analyses were performed using consistent regions of interest and standardized analysis parameters across all treatment groups to ensure comparability.

### 2.7. MAPK Inhibitor Pretreatment Experiments

To investigate the involvement of mitogen-activated protein kinase (MAPK) signaling pathways in apoptosis induced by combined curcumin and doxorubicin treatment, specific pharmacological inhibitors were used. HOS cells were pretreated with the ERK inhibitor PD98059 (25 μM), the p38 MAPK inhibitor SB203580 (25 μM), or the JNK inhibitor SP600125 (25 μM) for 1 h prior to drug treatment. Following inhibitor pretreatment, cells were treated with curcumin (9 μM) and doxorubicin (0.2 μM), either alone or in combination, as indicated. Cells were then incubated under standard culture conditions for an additional 48 h. After treatment, cells were harvested, and total cellular proteins were extracted for subsequent Western blot analysis. The effects of MAPK inhibition on apoptosis were evaluated by assessing the expression level of cleaved poly(ADP-ribose) polymerase (PARP) using Western blotting, as described above. These experiments were performed to determine the functional contribution of individual MAPK pathways to apoptosis induced by combined curcumin and doxorubicin treatment.

### 2.8. TXNDC5 Overexpression and Cell Transfection

TXNDC5 overexpression was achieved by plasmid transfection in HOS cells. One day prior to transfection, cells were seeded in culture dishes to reach approximately 80% confluence at the time of transfection. A TXNDC5 overexpression plasmid was used, with an empty vector serving as the control. Transfections were performed using X-tremeGENE™ HP DNA Transfection Reagent (Roche, Indianapolis, IN, USA) according to the manufacturer’s instructions. Briefly, serum-free culture medium was added to 1.5 mL microcentrifuge tubes, followed by the addition of X-tremeGENE™ HP reagent at a ratio of 3 μL per 1 μg of plasmid DNA. Plasmid DNA was then added at a ratio of 1 μg per transfection reaction, and the mixture was gently mixed and incubated at room temperature for 15 min to allow formation of DNA–reagent complexes. The complexes were subsequently added dropwise to cells cultured in complete medium. After transfection, cells were incubated at 37 °C in a humidified atmosphere containing 5% CO_2_. Cells were harvested 24 h after transfection for protein extraction and Western blot analysis to confirm TXNDC5 overexpression. Successfully transfected cells were then used for subsequent functional assays, including apoptosis analysis following combined curcumin and doxorubicin treatment.

### 2.9. Statistical Analysis

All quantitative data are presented as the mean ± standard deviation (SD) from at least three independent experiments. Statistical analyses were performed using GraphPad Prism software (version 9.0; GraphPad Software, San Diego, CA, USA). Comparisons between two groups were conducted using Student’s *t*-test. For comparisons among multiple groups, one-way analysis of variance (ANOVA) followed by Tukey’s post hoc test was applied. The assumptions of normality and homogeneity of variance were considered to be satisfied based on the experimental design and comparable variance observed among groups. A *p*-value of less than 0.05 was considered statistically significant.

## 3. Results

### 3.1. Effects of Curcumin and Doxorubicin, Alone and in Combination, on Cell Viability

**Figure 1:**
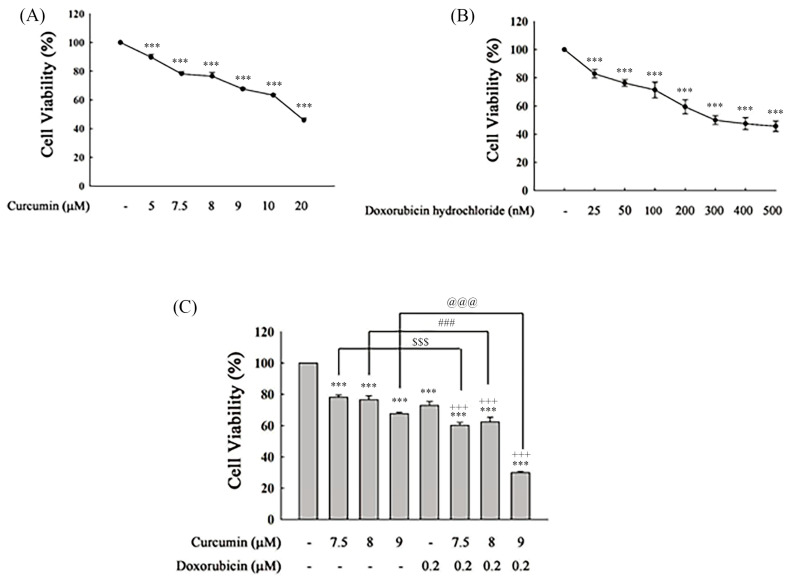
Effects of curcumin and doxorubicin on cell viability in HOS cells. (**A**,**B**) HOS cells were treated with increasing concentrations of curcumin (5–20 μM) or doxorubicin hydrochloride (25–500 nM) for 48 h, and cell viability was assessed using the CCK-8 assay. (**C**) Cells were treated with curcumin (7.5, 8, and 9 μM) alone or in combination with doxorubicin (0.2 μM) for 48 h. Cell viability was expressed as a percentage relative to the untreated control. Data are presented as mean ± SD from three independent experiments. Statistical analysis was performed using one-way ANOVA followed by Tukey’s post hoc test. *** *p* < 0.001 vs. untreated control; +++ *p* < 0.001 vs. doxorubicin alone; $$$, ###, and @@@ *p* < 0.001 indicate comparisons between curcumin-alone and combination treatment groups, as indicated.

Curcumin and doxorubicin individually reduced cell viability in a dose-dependent manner in HOS cells. As shown in Figure 1A, treatment with curcumin at concentrations ranging from 5 to 20 μM significantly decreased cell viability from approximately 100% in the control group to about 80%, 75%, 70%, 65%, and 45%, respectively. Similarly, doxorubicin hydrochloride treatment (25–500 nM) resulted in a progressive reduction in cell viability, decreasing from approximately 100% to about 80%, 75%, 70%, 60%, 50%, 45%, and 45% across the indicated concentrations (Figure 1B). Based on the dose–response profiles, sub-cytotoxic concentrations of curcumin (7.5, 8, and 9 μM) and doxorubicin (0.2 μM) were selected for combination treatment. The sub-cytotoxic concentrations refer to doses that result in less than approximately 20–40% reduction in cell viability. As shown in Figure 1C, treatment with curcumin alone reduced cell viability to approximately 78%, 72%, and 68% at 7.5, 8, and 9 μM, respectively, whereas doxorubicin (0.2 μM) alone resulted in a viability of approximately 75%. Notably, combined treatment further decreased cell viability to approximately 60%, 55%, and 30% at the corresponding curcumin concentrations, respectively, indicating a markedly enhanced inhibitory effect compared with either single treatment. The inhibitory effect of the combination treatment was more pronounced at higher curcumin concentrations, suggesting a concentration-dependent enhancement of growth suppression under combined treatment conditions. These results indicate that the combination treatment leads to a greater reduction in cell viability than either agent alone under comparable conditions.

### 3.2. Morphological Changes in HOS Cells Following Treatment with Curcumin and Doxorubicin

**Figure 2:**
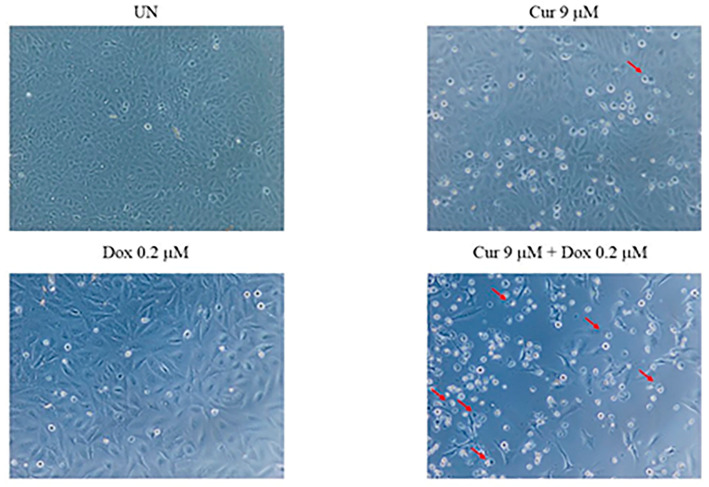
Morphological changes of HOS cells following treatment with curcumin and doxorubicin. Representative phase-contrast microscopic images of HOS cells under different treatment conditions. Cells were untreated (UN), treated with curcumin (9 μM), treated with doxorubicin (0.2 μM), or treated with a combination of curcumin (9 μM) and doxorubicin (0.2 μM) for 48 h. Red arrows indicate cytoplasmic vacuolization and morphological features associated with apoptotic cell death. Images were captured using an optical microscope.

To examine the morphological effects of curcumin and doxorubicin on HOS cells, phase-contrast microscopy was used to observe cellular changes following different treatments. As shown in Figure 2, untreated cells exhibited a typical spindle-shaped morphology with intact cell–cell adhesion. Treatment with curcumin (9 μM) or doxorubicin (0.2 μM) alone resulted in moderate morphological alterations, including cell rounding and the appearance of cytoplasmic vacuoles. In contrast, combined treatment with curcumin and doxorubicin induced more pronounced morphological changes, characterized by increased cell shrinkage, extensive vacuolization, and the presence of apoptotic-like features, as indicated by red arrows. These observations suggest that the combination treatment caused greater morphological disruption compared with either agent alone.

### 3.3. Curcumin Enhances Doxorubicin-Induced Apoptosis in HOS Cells

**Figure 3:**
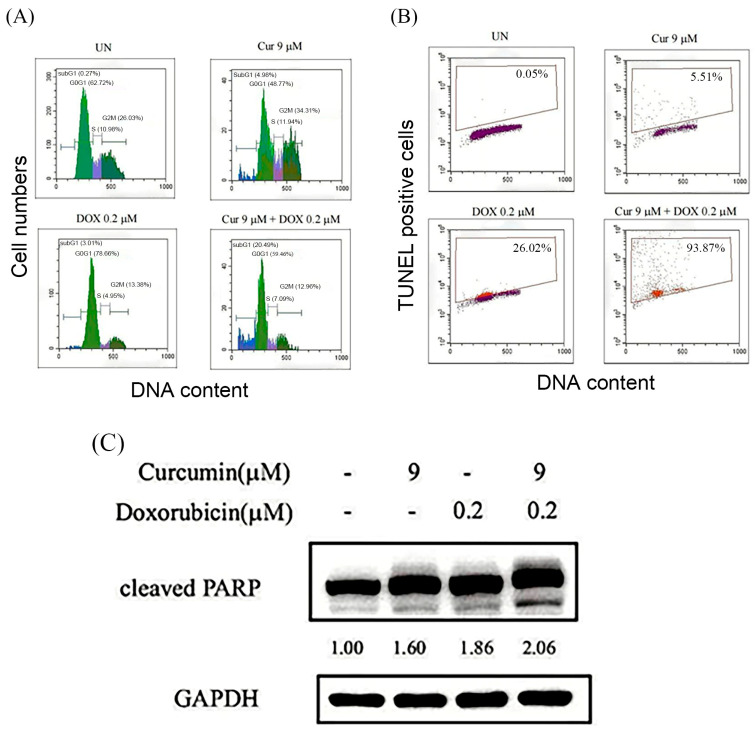
Effects of curcumin and doxorubicin on apoptosis induction in HOS cells. (**A**) Cell cycle distribution and sub-G1 population were analyzed by flow cytometry following propidium iodide (PI) staining after 48 h of treatment. HOS cells were left untreated (UN) or treated with curcumin (9 μM), doxorubicin (0.2 μM), or their combination. The sub-G1 fraction was used as an indicator of apoptotic cell death. (**B**) DNA fragmentation was assessed by TUNEL assay using an APO-BrdU™ TUNEL assay kit. Representative flow cytometric dot plots show TUNEL-positive cells, and percentages indicate the proportion of apoptotic cells. (**C**) Expression of apoptosis-associated protein cleaved PARP was analyzed by Western blotting following 48 h of treatment. GAPDH was used as a loading control. Relative protein expression levels were quantified by densitometric analysis.

Based on the observed reduction in cell viability following combined curcumin and doxorubicin treatment (Figure 1), together with apoptotic-like morphological changes revealed by phase-contrast microscopy (Figure 2), further analyses were performed to determine whether the enhanced growth inhibition was associated with increased and quantifiable apoptotic cell death. As shown in Figure 3A, flow cytometric analysis of propidium iodide (PI)-stained cells revealed a low sub-G1 population in untreated cells (0.27%). Treatment with curcumin (9 μM) or doxorubicin (0.2 μM) alone moderately increased the sub-G1 fraction to 4.98% and 3.01%, respectively. Notably, combined treatment with curcumin and doxorubicin resulted in a marked accumulation of sub-G1 cells, reaching 20.49%, indicating enhanced apoptotic cell death. Consistent with these findings, TUNEL assay demonstrated minimal DNA fragmentation in untreated cells (0.05%), whereas treatment with curcumin or doxorubicin alone increased the percentage of TUNEL-positive cells to 5.51% and 26.02%, respectively (Figure 3B). In contrast, combination treatment dramatically elevated the proportion of TUNEL-positive cells to 93.87%, indicating extensive DNA fragmentation under combined treatment conditions. Furthermore, Western blot analysis showed increased levels of cleaved PARP in cells treated with the combination of curcumin and doxorubicin compared with either agent alone (Figure 3C), further supporting the induction of apoptosis. Together, these results indicate that curcumin markedly potentiates doxorubicin-induced apoptotic responses in HOS cells.

### 3.4. Activation of p38 and JNK, but Not ERK, Contributes to Curcumin- and Doxorubicin-Induced Apoptosis in HOS Cells

**Figure 4:**
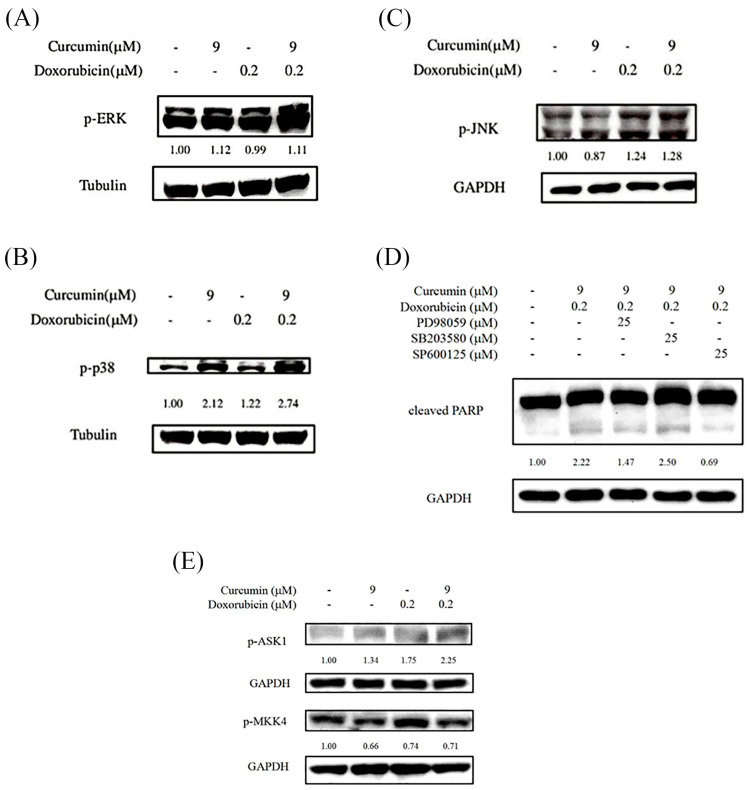
Involvement of MAPK signaling pathways in curcumin- and doxorubicin-induced apoptosis in HOS cells. HOS cells were treated with curcumin (9 μM), doxorubicin (0.2 μM), or their combination, as indicated. (**A**–**C**) The phosphorylation levels of mitogen-activated protein kinases (MAPKs), including extracellular signal-regulated kinase (p-ERK), p38 MAPK (p-p38), and c-Jun *N*-terminal kinase (p-JNK), were analyzed by Western blotting following 48 h of treatment and normalized to GAPDH. (**D**) The involvement of MAPK signaling pathways in apoptosis was further evaluated using specific MAPK inhibitors. Cells were pretreated with PD98059 (ERK inhibitor, 25 μM), SB203580 (p38 inhibitor, 25 μM), or SP600125 (JNK inhibitor, 25 μM) for 1 h prior to combined curcumin and doxorubicin treatment, and cleaved PARP expression was analyzed after 48 h to evaluate the functional involvement of MAPK signaling in apoptosis. (**E**) To investigate upstream signaling events involved in JNK activation, phosphorylation of apoptosis signal–regulating kinase 1 (p-ASK1) and mitogen-activated protein kinase kinase 4 (p-MKK4) was examined by Western blot analysis following 1 h of treatment.

To further elucidate the signaling pathways involved in apoptosis induced by combined curcumin and doxorubicin treatment, the activation status of major MAPK pathways was examined. As shown in Figure 4A–C, combined treatment markedly increased the phosphorylation levels of p38 MAPK and c-Jun *N*-terminal kinase (JNK) compared with untreated or single-agent-treated cells. In contrast, no apparent increase in extracellular signal-regulated kinase (ERK) phosphorylation was observed following the combination treatment. To assess the functional relevance of MAPK signaling in apoptosis induction, specific MAPK inhibitors were applied prior to combined drug treatment. As shown in Figure 4D, inhibition of ERK phosphorylation by PD98059 or inhibition of JNK phosphorylation by SP600125 significantly reduced the level of cleaved PARP induced by the combination of curcumin and doxorubicin. To further examine whether early JNK activation is a conserved response across osteosarcoma cell lines, JNK phosphorylation was also assessed in MG-63 cells. As shown in Supplementary Figure S3, combined curcumin and doxorubicin treatment induced a rapid increase in JNK phosphorylation within 1 h, supporting early activation of JNK signaling in response to combination treatment. In contrast, inhibition of p38 signaling by SB203580 did not attenuate cleaved PARP expression. These results suggest that JNK activation and ERK signaling, but not p38 activation, are involved in mediating apoptosis induced by combined curcumin and doxorubicin treatment in HOS cells. Given the prominent involvement of JNK signaling in apoptosis induction, upstream signaling events regulating JNK activation were further investigated. As shown in Figure 4E, phosphorylation of apoptosis signal–regulating kinase 1 (ASK1) was increased following combined curcumin and doxorubicin treatment at an early time point (1 h). In contrast, phosphorylation of mitogen-activated protein kinase kinase 4 (MKK4), a known upstream kinase of JNK, was not markedly altered under the same conditions. This apparent discrepancy suggests that phosphorylation status alone may not directly reflect functional involvement in apoptotic execution, and that distinct MAPK branches may differentially contribute to stress signaling and apoptosis under these conditions. These results reflect relative changes in phosphorylation levels under the indicated conditions rather than absolute activation of MAPK pathways.

### 3.5. TXNDC5 Downregulation Is Not Sufficient to Prevent Apoptosis Induced by Combined Curcumin and Doxorubicin Treatment

**Figure 5:**
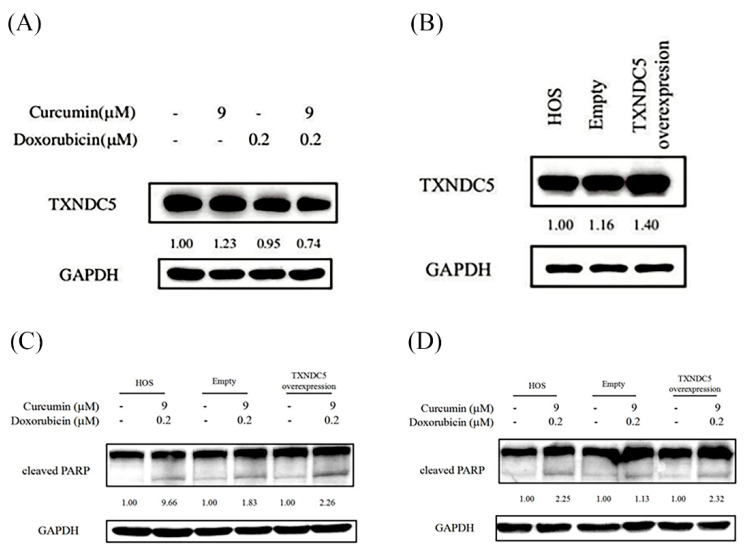
Role of TXNDC5 in modulating apoptosis induced by combined curcumin and doxorubicin treatment in HOS cells. (**A**) The protein expression level of thioredoxin domain-containing protein 5 (TXNDC5) was analyzed by Western blotting in HOS cells following treatment with curcumin (9 μM), doxorubicin (0.2 μM), or their combination, as indicated. (**B**) TXNDC5 expression was confirmed in parental, vector control, and TXNDC5-overexpressing HOS cells. (**C**,**D**) Parental HOS cells, empty vector–transfected cells, and TXNDC5-overexpressing cells were treated with curcumin (9 μM) and doxorubicin (0.2 μM) for 24 h or 48 h, followed by analysis of cleaved PARP expression by Western blotting. GAPDH was used as a loading control. Relative protein expression levels were quantified by densitometric analysis and are shown below the corresponding blots.

To investigate the role of TXNDC5 in apoptosis induced by combined curcumin and doxorubicin treatment, TXNDC5 protein expression was first examined. As shown in Figure 5A, treatment with the combination of curcumin and doxorubicin resulted in a reduction of TXNDC5 protein levels compared with untreated or single-agent-treated cells. Overexpression of TXNDC5 was confirmed by Western blot analysis in TXNDC5-overexpressing cells relative to parental HOS cells and empty vector–transfected controls (Figure 5B). Next, the effect of TXNDC5 overexpression on apoptosis induced by combined drug treatment was evaluated. As shown in Figure 5C,D, combined curcumin and doxorubicin treatment markedly increased cleaved PARP levels at both 24 h and 48 h. However, enforced overexpression of TXNDC5 did not attenuate cleaved PARP induction induced by the combination treatment at either time point. These results indicate that although TXNDC5 expression is downregulated following combined treatment, restoration of TXNDC5 expression is insufficient to suppress apoptosis induced by curcumin and doxorubicin in HOS cells.

### 3.6. Combined Curcumin and Doxorubicin Treatment Modulates ER Stress, Autophagy, and NF-κB–Associated Signaling

**Figure 6:**
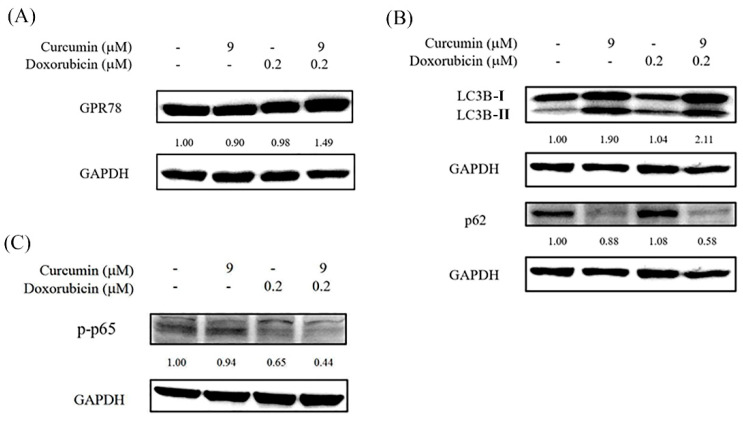
Combined curcumin and doxorubicin treatment modulates ER stress-, autophagy-, and NF-κB–associated signaling pathways in HOS cells. HOS cells were treated with curcumin (9 μM), doxorubicin (0.2 μM), or their combination, as indicated, for 48 h. (**A**) GRP78 expression was analyzed as a marker of ER stress. (**B**) Autophagy-associated markers LC3B and p62 were analyzed by Western blotting. (**C**) The phosphorylation of NF-κB p65 was analyzed to assess NF-κB signaling activity. GAPDH was used as a loading control. Relative protein expression levels were quantified by densitometric analysis.

In addition to MAPK-dependent apoptotic signaling, the effects of combined curcumin and doxorubicin treatment on other stress-associated pathways were further examined. As shown in Figure 6A, expression of glucose-regulated protein 78 (GRP78), a key marker of endoplasmic reticulum (ER) stress, was increased following combined treatment compared with untreated or single-agent-treated cells. Autophagy-associated markers were also evaluated. As shown in Figure 6B, combined treatment resulted in increased levels of LC3B-II accompanied by a reduction in p62 expression, consistent with alterations in autophagy-associated protein markers. In parallel, the effect of combined treatment on nuclear factor kappa B (NF-κB) signaling was assessed. As shown in Figure 6C, phosphorylation of NF-κB p65 was reduced following combined curcumin and doxorubicin treatment. Together, these findings suggest that combined treatment is associated with concurrent modulation of ER stress, autophagy-related responses, and NF-κB signaling in HOS cells.

### 3.7. Validation of the Inhibitory Effect of Combined Curcumin and Doxorubicin in MG-63 Osteosarcoma Cells

To evaluate whether the growth-inhibitory effect of combined curcumin and doxorubicin treatment was reproducible in another osteosarcoma cell model, MG-63 cells were subjected to cell viability analysis. As shown in Supplementary Figure S1, treatment with curcumin or doxorubicin alone moderately reduced MG-63 cell viability after 48 h. Notably, combined treatment with curcumin (15 μM) plus doxorubicin (0.2 μM), as well as curcumin (15 μM) plus doxorubicin (0.3 μM), resulted in a more pronounced reduction in cell viability compared with either agent alone. These findings indicate that the enhanced inhibitory effect of combined curcumin and doxorubicin treatment is not restricted to HOS cells but can also be observed in MG-63 osteosarcoma cells, supporting the generalizability of the combination effect across osteosarcoma cell lines. To further determine whether the enhanced growth inhibition observed in MG-63 cells was associated with apoptotic cell death, the expression of cleaved poly(ADP-ribose) polymerase (PARP) was examined by Western blot analysis. To allow clearer detection of apoptotic signaling, MG-63 cells were treated with curcumin (15 μM), doxorubicin (0.3μM), or the indicated combinations for 48 h. As shown in Supplementary Figure S2, treatment with either curcumin or doxorubicin alone resulted in modest induction of cleaved PARP. In contrast, combined treatment with curcumin (15 μM) plus doxorubicin (0.3 μM) led to a more pronounced increase in cleaved PARP levels. Consistent with the viability data, combined curcumin and doxorubicin treatment also enhanced apoptotic signaling in MG-63 cells, as evidenced by increased cleaved PARP expression under combination conditions (Supplementary Figure S2).

## 4. Discussion

Consistent with the concept that curcumin can function as a chemosensitizing phytochemical, studies published since 2021 have demonstrated that curcumin enhances the anticancer response to doxorubicin through modulation of stress-adaptive and survival-related pathways, although the dominant molecular nodes appear to vary across tumor contexts. For instance, in doxorubicin-insensitive breast cancer models, curcumin has been reported to restore doxorubicin responsiveness primarily by targeting Aurora A–centered signaling networks and drug-resistance programs [33], rather than directly implicating MAPK signaling as the central mediator of the synergistic effect. In contrast, accumulating mechanistic evidence has highlighted the c-Jun *N*-terminal kinase (JNK) cascade as a key stress-activated pathway through which curcumin promotes tumor cell death under specific cellular conditions, including oxidative stress–driven apoptosis or regulated cell death [28,34,35,36].

Building on this framework, our data suggest that JNK activation represents a principal execution-associated MAPK signal in human osteosarcoma HOS cells subjected to combined curcumin and doxorubicin treatment. This conclusion is supported by the marked increase in JNK phosphorylation and by the ability of the JNK inhibitor SP600125 to significantly attenuate PARP cleavage (Figure 4), indicating a functional requirement for JNK signaling in apoptotic execution.

Interestingly, although combined curcumin and doxorubicin treatment elicited a pronounced increase in p38 MAPK phosphorylation (Figure 4B), pharmacological inhibition of p38 signaling using SB203580 failed to attenuate PARP cleavage induced by the combination treatment (Figure 4D). This dissociation between p38 activation and apoptotic execution suggests that p38 MAPK activation in this context is not functionally required for apoptosis induction. Instead, p38 activation may reflect a stress-responsive or adaptive signaling event triggered by chemotherapeutic and phytochemical stress, rather than serving as a dominant execution pathway. Indeed, p38 MAPK has been reported to exert context-dependent functions in cancer cells, acting either as a pro-apoptotic mediator or as a regulator of stress adaptation and survival depending on cellular context and stimulus intensity [37]. In several models, p38 activation accompanies cellular stress without being essential for downstream apoptotic execution once stronger death-promoting pathways are engaged [38,39,40].

In the present study, our data support a model in which p38 MAPK activation represents an upstream stress-sensing response to combined curcumin and doxorubicin treatment, whereas JNK signaling functions as the principal execution-associated MAPK pathway driving apoptotic cell death. Notably, although ERK phosphorylation was not elevated by the combination treatment, pharmacological inhibition of ERK signaling nevertheless reduced cleaved PARP expression (Figure 4). This finding suggests that basal ERK activity may function in a permissive or cooperative manner to support JNK-dependent apoptotic signaling, rather than acting as an inducible driver of apoptosis under these experimental conditions.

It should be noted that phosphorylation levels of MAPK proteins were normalized to the loading control (GAPDH) rather than to their corresponding total protein levels. Although normalization to total protein is commonly applied in signaling studies, the consistent phosphorylation patterns observed across independent experiments, together with functional validation using pharmacological inhibitors (e.g., SP600125), support the interpretation of MAPK pathway involvement. Nevertheless, the present findings should be interpreted as relative changes in phosphorylation status rather than definitive evidence of absolute pathway activation.

Mechanistically, upstream signaling analysis further revealed that combination treatment rapidly increased ASK1 phosphorylation, whereas MKK4 phosphorylation was not detectably increased within the same early time window. This pattern suggests that ASK1-to-JNK transmission may proceed through alternative intermediates (e.g., preferential engagement of other MAP2Ks such as MKK7, or transient kinetics of MKK4 activation that fall outside the sampling window), and highlights that ASK1 activation alone is not necessarily mirrored by a sustained p-MKK4 signal at the time point examined. Such a “non-parallel” phosphorylation pattern is compatible with the broader view that JNK signaling is highly stimulus-, timing-, and scaffold-dependent, and that upstream kinase readouts can be pathway-selective rather than uniformly propagated across all canonical nodes. Collectively, compared with recent chemosensitization studies emphasizing resistance networks [27,41], our findings add a complementary mechanistic angle by identifying JNK-dependent MAPK signaling as the dominant apoptosis-associated axis under curcumin–doxorubicin co-treatment in osteosarcoma cells, while also distinguishing inducible MAPK signals from permissive basal signaling requirements.

TXNDC5 is known to play a role in redox homeostasis and cellular stress responses, and its dysregulation has been implicated in cancer progression and therapeutic resistance [22,42]. In the present study, combined curcumin and doxorubicin treatment reduced TXNDC5 expression in HOS cells. However, enforced overexpression of TXNDC5 failed to attenuate PARP cleavage induced by the combination treatment, indicating that TXNDC5 downregulation alone is not sufficient to account for apoptotic execution. One possible explanation is that apoptosis induced by the combined treatment is driven by dominant pro-apoptotic signaling pathways that override TXNDC5-mediated protective mechanisms. Consistent with this notion, our data suggest that JNK activation is involved in apoptosis induced by combined curcumin and doxorubicin treatment, indicating that MAPK-dependent apoptotic signaling may predominate over the modulatory effects of TXNDC5. In addition, TXNDC5 may function at an upstream stress-response level, whereas PARP cleavage represents a late-stage apoptotic event. Once the execution phase of apoptosis has been initiated, restoration of TXNDC5 expression may be insufficient to reverse apoptotic progression. This temporal dissociation between stress-associated regulators and apoptotic execution factors has also been described in other models of chemotherapeutic stress. Taken together, these findings suggest that TXNDC5 downregulation is associated with, but not determinative of, apoptosis induced by combined curcumin and doxorubicin treatment. Rather than acting as a central apoptotic regulator, TXNDC5 may serve as a stress-responsive modulator whose effects may be secondary to stronger pro-apoptotic signaling pathways activated by the combination treatment. Consistent with this interpretation, TXNDC5 downregulation accompanies combined curcumin and doxorubicin treatment but does not appear sufficient to counteract apoptosis associated with MAPK-dependent signaling.

While the present findings support the involvement of MAPK signaling in apoptosis induced by combined curcumin and doxorubicin treatment, the mechanistic relationships between individual MAPK pathways, TXNDC5 regulation, and apoptotic execution should be interpreted with caution. Although JNK phosphorylation and inhibitor studies suggest a functional role in apoptosis, the current data do not establish a direct causal relationship at the molecular level. Similarly, although TXNDC5 expression was reduced following treatment, its precise role in regulating apoptotic signaling remains to be further elucidated. Therefore, these findings primarily indicate an association rather than definitive mechanistic linkage.

In addition to JNK-dependent MAPK signaling, our observation that GRP78 was increased by the combination treatment suggests engagement of an endoplasmic reticulum (ER) stress response, which is a common cellular reaction to proteotoxic and oxidative insults induced by chemotherapy. ER stress has been reported to play context-dependent roles in cancer cells, functioning as either a protective mechanism or a contributor to cell death depending on stress intensity and duration [43,44]. Importantly, curcumin has been reported to enhance chemosensitivity by amplifying ER stress signaling in cancer cells. For example, curcumin was shown to increase cisplatin sensitivity in non-small cell lung cancer cells through activation of ER stress–associated pathways, supporting the concept that ER stress augmentation can contribute to curcumin-mediated chemosensitization [45]. Moreover, mechanistic evidence indicates that curcumin itself can trigger ER stress programs (e.g., ATF6-associated signaling) and promote apoptotic execution, providing a plausible basis for why ER stress markers may rise in our co-treatment setting [46]. Consistently, ER stress activation has also been observed alongside stress-activated kinase signaling (including JNK/p38) in curcumin-treated cancer models, suggesting that ER stress may occur as part of a broader stress-integrated response rather than as an isolated pathway [47,48]. In the present study, because GRP78 was assessed as a marker without functional interrogation of unfolded protein response (UPR) branches (PERK/eIF2α/ATF4–CHOP, IRE1α/XBP1, or ATF6) or ER stress dependence, the current data support ER stress engagement as an accompanying stress signature rather than definitive evidence that ER stress is required for apoptosis. Future experiments using pathway-level readouts (e.g., CHOP induction, XBP1 splicing, ATF6 cleavage) and pharmacologic or genetic modulation would be needed to determine whether ER stress is mechanistically upstream of the JNK-dependent apoptotic axis observed here.

Furthermore, the interpretation of increased LC3B-II levels accompanied by reduced p62 expression requires careful consideration, as autophagy is well recognized to exert context-dependent roles in cancer cells under chemotherapeutic or phytochemical stress. In many settings, autophagy is activated as an adaptive or compensatory response that promotes cell survival by alleviating proteotoxic and metabolic stress; however, under conditions of sustained or excessive stress, autophagy may also cooperate with apoptotic signaling or contribute to cell death [49,50]. In the present study, the observed alterations in autophagy-associated markers most plausibly reflect engagement of a stress-responsive autophagy program triggered by combined curcumin and doxorubicin treatment, rather than definitive evidence that autophagy directly drives apoptotic execution.

Consistent with this interpretation, accumulating evidence indicates that curcumin can modulate autophagy through multiple signaling nodes, including the PI3K/Akt/mTOR axis, and that the functional outcome of curcumin-induced autophagy is highly dependent on tumor type, cellular context, and stress intensity [28]. Recent studies have reported that curcumin alone or in combination with conventional chemotherapeutic agents can enhance autophagy-associated responses in cancer cells, often in parallel with apoptotic signaling, supporting the notion that autophagy activation frequently accompanies chemosensitization rather than acting as an isolated death mechanism [45,47]. Nevertheless, because autophagic flux and functional dependence on autophagy were not directly assessed in this study—such as by using lysosomal inhibitors, tandem fluorescent LC3 reporters, or genetic disruption of core autophagy regulators—the present findings should be interpreted as indicative of autophagy modulation as part of a broader cellular stress landscape. Future studies will be required to determine whether autophagy serves a protective, neutral, or contributory role in the apoptotic response elicited by combined curcumin and doxorubicin treatment.

In parallel, changes in additional stress- and survival-related signaling pathways were also observed. Specifically, suppression of NF-κB p65 phosphorylation was observed following combined treatment (Figure 6C). Given the established role of NF-κB signaling in promoting cell survival and chemoresistance, attenuation of NF-κB activity may further contribute to a cellular environment that favors apoptotic signaling. However, as NF-κB signaling was not functionally interrogated in this study, its precise contribution to apoptosis induction warrants further investigation.

In addition to MAPK-dependent apoptotic signaling, accumulating evidence suggests that curcumin-mediated chemosensitization is closely associated with the induction of oxidative stress and disruption of redox homeostasis. Recent studies have demonstrated that combined curcumin and doxorubicin treatment markedly increases intracellular reactive oxygen species (ROS) levels, leading to mitochondrial dysfunction, ATP depletion, cytochrome c release, and caspase-3 activation. This oxidative stress–driven cytotoxicity is further accompanied by iron accumulation and lipid peroxidation, ultimately contributing to ferroptosis-associated cell death pathways. Importantly, pharmacological inhibition of ROS or ferroptosis has been shown to attenuate apoptosis and DNA fragmentation, supporting a mechanistic link between oxidative stress, ferroptosis, and apoptotic signaling [51]. These findings provide a broader mechanistic framework in which oxidative stress acts as an upstream trigger that integrates multiple stress-response pathways, including MAPK activation, ER stress, and autophagy modulation. In this context, our data support a model in which curcumin–doxorubicin co-treatment induces a multifaceted stress response, while JNK-dependent MAPK signaling serves as the dominant execution-associated pathway driving apoptosis in osteosarcoma cells. Other stress-related events, such as ER stress activation and autophagy-associated changes, may reflect downstream or parallel adaptive responses to oxidative stress rather than primary determinants of apoptotic execution.

Although the p-JNK signal observed in MG-63 cells appeared relatively weaker, the increase in phosphorylation following combined treatment was consistently detected across independent experiments. This variation may reflect differences in protein expression levels or antibody sensitivity between cell lines. These findings provide supportive validation that JNK activation is not restricted to HOS cells, although the mechanistic conclusions of the present study are primarily based on HOS cells.

It is also important to consider the intrinsic differences between osteosarcoma cell lines used in this study. HOS and MG-63 cells differ in their genetic background, differentiation status, and tumorigenic potential. MG-63 cells are generally considered to exhibit a more immature osteoblastic phenotype and distinct proliferative and signaling characteristics compared with HOS cells. These intrinsic differences may influence cellular stress responses and downstream signaling activation, including MAPK pathways. In this context, while MG-63 cells were used to provide supportive validation of the combination effect, the detailed mechanistic findings in the present study are primarily based on HOS cells. Therefore, variations observed between cell lines may reflect cell type-specific signaling properties rather than inconsistencies in experimental outcomes.

**Figure 7:**
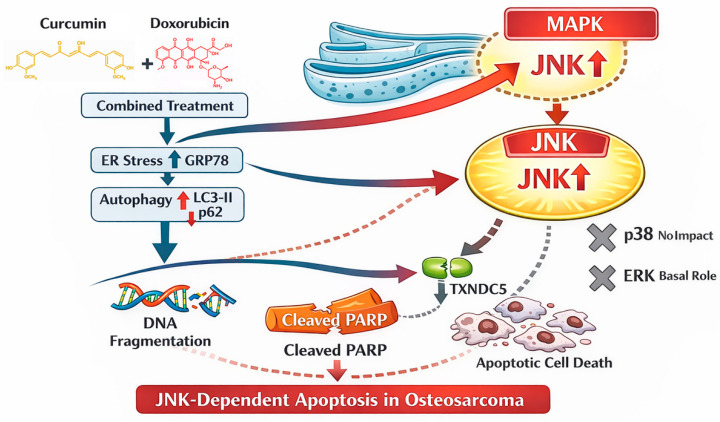
Schematic model illustrating that combined curcumin and doxorubicin treatment induces apoptosis predominantly through JNK-dependent MAPK signaling, with ER stress, autophagy, NF-κB modulation, and TXNDC5 downregulation as supportive or context-dependent responses.

Collectively, these observations suggest that combined curcumin and doxorubicin treatment induces a broad cellular stress response involving MAPK signaling, ER stress, autophagy-associated processes, and NF-κB modulation. Among these pathways, JNK-dependent MAPK signaling represents the dominant and functionally required axis mediating apoptotic execution, while other stress-response pathways may serve supportive or context-dependent functions. A schematic model summarizing these findings is presented in Figure 7, highlighting JNK activation as the principal execution-associated pathway, with ER stress, autophagy, NF-κB modulation, and TXNDC5 downregulation representing accompanying stress responses. By distinguishing the primary apoptotic driver from these associated pathways, this study provides mechanistic insight into curcumin-mediated chemosensitization and supports the potential application of curcumin as an adjuvant to enhance the therapeutic efficacy of doxorubicin in osteosarcoma.

## 5. Conclusions

This study demonstrates that combined curcumin and doxorubicin treatment effectively induces apoptosis that appears to be primarily associated with JNK-dependent MAPK signaling in human osteosarcoma cells. The enhanced anticancer effect is characterized by reduced cell viability and activation of apoptotic markers, including sub-G1 accumulation, DNA fragmentation, and PARP cleavage. Mechanistically, this apoptotic response is functionally dependent on JNK activation, as pharmacological inhibition of JNK attenuated PARP cleavage. In contrast, p38 activation and ERK signaling were not required as direct apoptotic drivers. Although TXNDC5 expression was reduced following combined treatment, TXNDC5 overexpression failed to suppress apoptosis, indicating a non-determinative role. Collectively, these findings support curcumin as a potential adjuvant agent that enhances doxorubicin-induced apoptosis through JNK-dependent signaling in osteosarcoma cells.

## Data Availability

The data that support the findings of this study are contained within the article.

## Supplementary Materials

The following supporting information can be downloaded at: https://www.mdpi.com/article/10.3390/nu18081235/s1, Figure S1: Effects of combined curcumin and doxorubicin treatment on cell viability in MG-63 cells; Figure S2: Induction of apoptosis in MG-63 cells assessed by cleaved PARP expression; Figure S3: Activation of JNK signaling in MG-63 cells following combination treatment.
