# Supplementary material for: Combined Curcumin and Doxorubicin Induce Apoptosis via JNK-Dependent MAPK Signaling Independent of TXNDC5 in Human Osteosarcoma Cells
Source: Nutrients. 2026 Apr 14;18(8):1235. doi: 10.3390/nu18081235 (PMC13119041; doi:10.3390/nu18081235)
Supplement: Supplementary file 1 [file nutrients-18-01235-s001.zip › nutrients-4199008-supplementary.pdf]

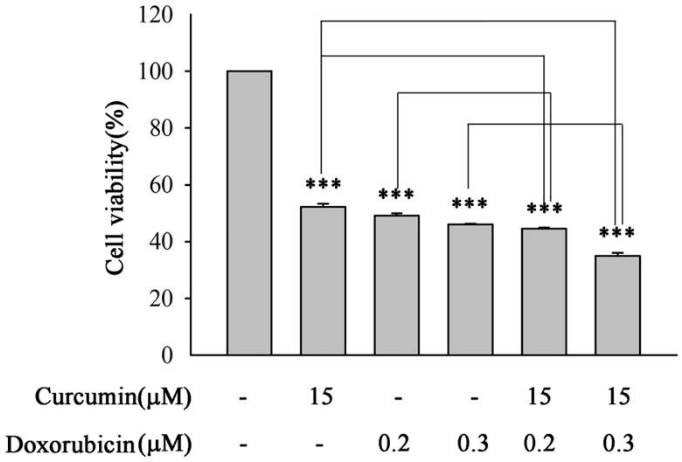

Figure S1. Effects of combined curcumin and doxorubicin treatment on cell viability in MG-63 osteosarcoma cells. MG-63 cells were treated with curcumin (15 μM), doxorubicin (0.2, or 0.3 μM), or the indicated combinations for 48 h. Cell viability was assessed using the CCK-8 assay and expressed as a percentage relative to the untreated control. Data are presented as mean ± SD from three independent experiments. *** $p < 0.001$ compared with the untreated control. Statistical comparisons between combination treatment groups and the corresponding single-agent treatments are indicated in the figure. Statistical comparisons between combination treatment groups and the corresponding single-agent treatments highlight enhanced growth inhibition at selected combination doses.

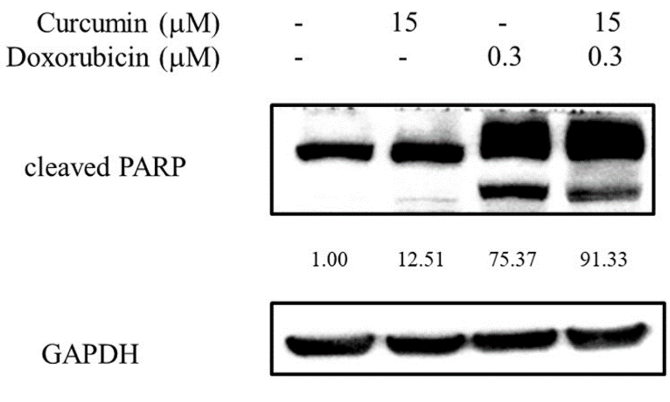

Figure S2. Induction of apoptosis by combined curcumin and doxorubicin treatment in MG-63 osteosarcoma cells. MG-63 cells were treated with curcumin (Cur; 15 μM), doxorubicin hydrochloride (Dox; 0.3μM), or the indicated combinations for 48 h. Total cellular proteins were extracted and cleaved PARP expression was analyzed by Western blotting. GAPDH was used as a loading control. Relative protein expression levels were quantified by densitometric analysis and are shown below the corresponding blots.

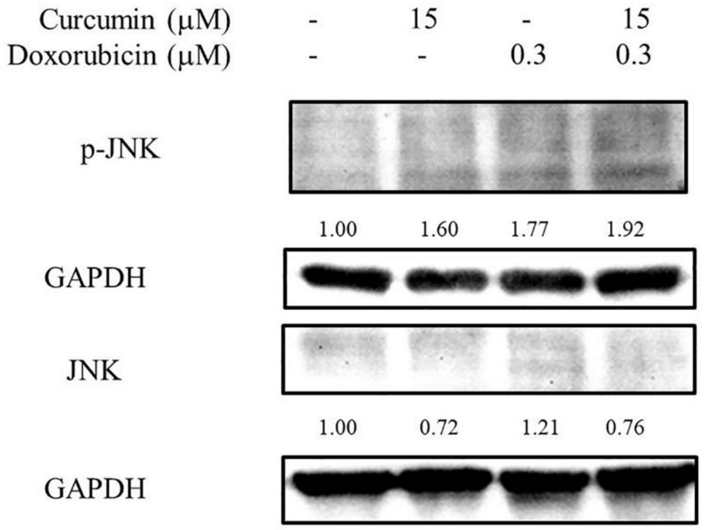

Figure S3. Activation of JNK signaling by combined curcumin and doxorubicin treatment in MG-63 osteosarcoma cells. MG-63 cells were treated with curcumin (Cur; 15 μM), doxorubicin (Dox; 0.3 μM), or the indicated combinations for 1 h. Total cellular proteins were extracted, and the expression levels of phosphorylated JNK (p-JNK) and total JNK were analyzed by Western blotting. GAPDH was used as a loading control. Relative protein expression levels were quantified by densitometric analysis and are presented below the corresponding immunoblots.
